# SARS-CoV-2 infection and vaccination trigger long-lived B and CD4^+^ T lymphocytes with implications for booster strategies

**DOI:** 10.1172/JCI157990

**Published:** 2022-03-15

**Authors:** Alessio Mazzoni, Anna Vanni, Michele Spinicci, Giulia Lamacchia, Seble Tekle Kiros, Arianna Rocca, Manuela Capone, Nicoletta Di Lauria, Lorenzo Salvati, Alberto Carnasciali, Elisabetta Mantengoli, Parham Farahvachi, Lorenzo Zammarchi, Filippo Lagi, Maria Grazia Colao, Francesco Liotta, Lorenzo Cosmi, Laura Maggi, Alessandro Bartoloni, Gian Maria Rossolini, Francesco Annunziato

**Affiliations:** 1 Department of Experimental and Clinical Medicine, University of Florence, Florence, Italy.; 2 Infectious and Tropical Diseases Unit,; 3 Microbiology and Virology Unit,; 4 Immunology and Cell Therapy Unit, and; 5 Flow Cytometry Diagnostic Center and Immunotherapy, Careggi University Hospital, Florence, Italy.

**Keywords:** COVID-19, Adaptive immunity, Cellular immune response

## Abstract

**BACKGROUND:**

Immunization against SARS-CoV-2, the causative agent of COVID-19, occurs via natural infection or vaccination. However, it is currently unknown how long infection- or vaccination-induced immunological memory will last.

**METHODS:**

We performed a longitudinal evaluation of immunological memory to SARS-CoV-2 up to 1 year after infection and following mRNA vaccination in naive individuals and individuals recovered from COVID-19 infection.

**RESULTS:**

We found that memory cells are still detectable 8 months after vaccination, while antibody levels decline significantly, especially in naive individuals. We also found that a booster injection is efficacious in reactivating immunological memory to spike protein in naive individuals, whereas it was ineffective in previously SARS-CoV-2–infected individuals. Finally, we observed a similar kinetics of decay of humoral and cellular immunity to SARS-CoV-2 up to 1 year following natural infection in a cohort of unvaccinated individuals.

**CONCLUSION:**

Short-term persistence of humoral immunity, together with the reduced neutralization capacity versus the currently prevailing SARS-CoV-2 variants, may account for reinfections and breakthrough infections. Long-lived memory B and CD4^+^ T cells may protect from severe disease development. In naive individuals, a booster dose restored optimal anti-spike immunity, whereas the needs for vaccinated individuals who have recovered from COVID-19 have yet to be defined.

**FUNDING:**

This study was supported by funds to the Department of Experimental and Clinical Medicine, University of Florence (Project Excellence Departments 2018–2022), the University of Florence (project RICTD2122), the Italian Ministry of Health (COVID-2020-12371849), and the region of Tuscany (TagSARS CoV 2).

## Graphical abstract

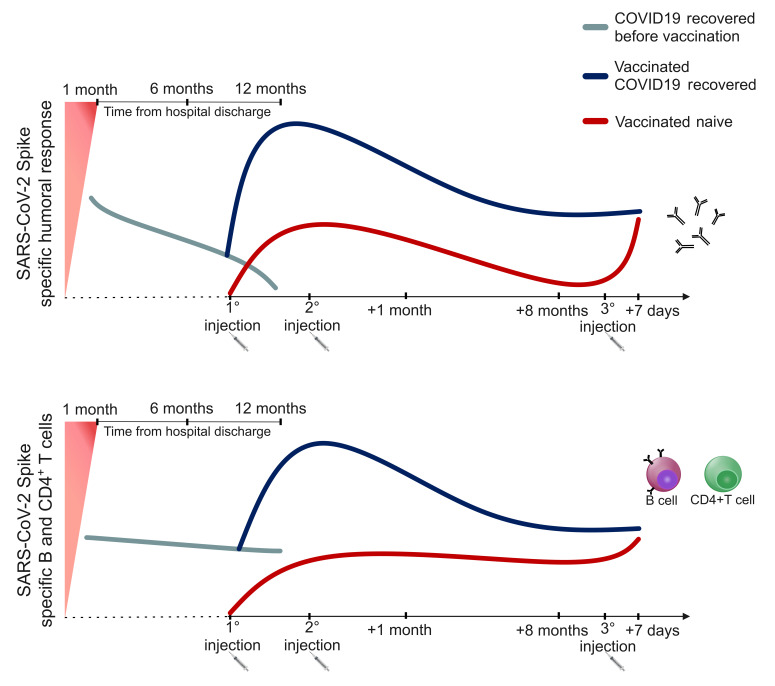

## Introduction

Immunization against SARS-CoV-2, the causative agent of COVID-19 occurs via natural infection or vaccination. As of December 20, 2021, more than 273 million people have been infected worldwide, with more than 5 million deaths (1). At least 1 vaccine dose has been administered to more than 4 billion people, and 3.49 billion people have been fully vaccinated, with considerable differences in vaccination rates between high- and low-income countries (1). In order to contain the COVID-19 pandemic, achieving high immunization levels worldwide is necessary. However, it is currently unknown how long infection- or vaccination-induced immunological memory will last. Regarding natural infection, it has been shown that, although antibody levels decline, memory B and T cells can be detected up to 8 months after recovery in most individuals (2), although 10% to 20% of them display a weak immunological memory (2, 3). The magnitude of immunological memory to SARS-CoV-2 directly correlates to disease severity, as asymptomatic individuals show reduced levels of virus-specific immunity, both in the acute and memory phase (4, 5). Information about longevity of vaccine-induced immunological memory is even scarcer, as the immunization campaign started less than 1 year ago. Real-world studies have shown that 6 months following the complete mRNA vaccination cycle, protection against COVID-19 is significantly reduced in all age groups, although protection from severe disease and hospitalization remains high (6–9). This phenomenon may be the result of progressive waning of immune protection. Nonetheless, the occurrence of SARS-CoV-2 variants, such as Delta (B.1.617.2) and, more recently, Omicron (B.1.1.529), that show increased transmission capability and immune evasion potential also plays a relevant role. These data raised the question about the need for a booster dose administration, and many countries are now recommending a third vaccine injection 6 months following the second dose. However, it is still not clear if a third injection is required in all individuals, or if it should be reserved to specific groups based on higher professional exposure risk or increased susceptibility to COVID-19 due to concomitant diseases and corresponding treatment regimens. To further complicate this scenario, it has been shown that the second mRNA vaccine injection is dispensable in individuals with previous SARS-CoV-2 infection, because the first administration is sufficient to maximize their immune response to spike (3, 10). Vaccine delivery to individuals who have recovered from COVID-19 results in the so-called “hybrid immunity,” characterized by increased strength of humoral and cellular response and increased breadth of antibody repertoire compared with individuals with natural infection or vaccination in naive individuals (11).

In this work, we performed a longitudinal evaluation of immunological memory to SARS-CoV-2 up to 8 months following mRNA vaccination in naive individuals and individuals who have recovered from COVID-19 infection. We also assessed the immunological effects of a booster injection in a cohort of individuals from both groups. Our results show that, despite a progressive decline affecting mainly antibody levels, humoral and cellular responses are still detectable in naive individuals and individuals who have recovered from COVID-19 8 months after vaccination. Moreover, we observed that a booster injection is more efficacious in reactivating immunological memory to spike in naive individuals than in previously SARS-CoV-2–infected individuals. Finally, we also assessed the longevity of immunological memory to SARS-CoV-2 following natural infection up to 1 year in a cohort of unvaccinated individuals. In this case, despite a decline in antibody levels, B and T cells memory responses were still detectable 12 months after infection.

## Results

### Anti-spike antibodies decline more rapidly following vaccination in naive individuals than in individuals who have recovered from COVID-19.

This study follows up our previous work, in which we monitored the vaccine-induced humoral and cellular immune response against SARS-CoV-2 spike protein up to 1 week after the second vaccine injection in naive individuals and individuals who have recovered from COVID-19 (3). Here, in order to evaluate the duration of immunological memory induced by vaccination, we extended our cohort to 15 individuals per group who were longitudinally evaluated up to 8 months after vaccination.

Demographic and clinical characteristics of the 30 recruited individuals are detailed in Supplemental Table 1 (supplemental material available online with this article; https://doi.org/10.1172/JCI157990DS1). We obtained a blood draw at basal time (baseline, before injection of the first dose), after 21 days (before injection of the second dose), and then after 1, 6, and 8 months to monitor humoral response and the presence of SARS-CoV-2 spike-specific B and T cells.

**Figure 1:**
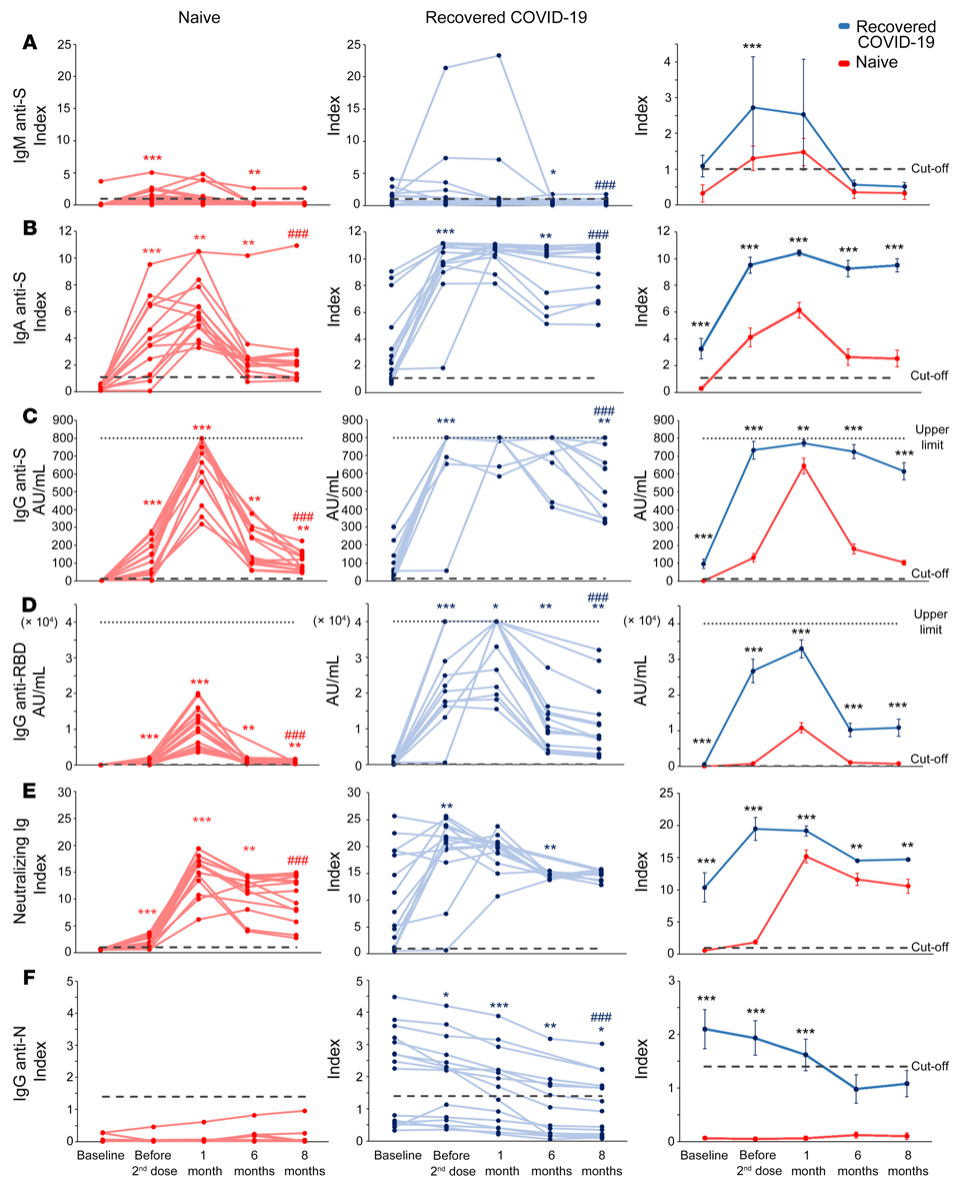
Longitudinal evaluation of anti-SARS-CoV-2 antibody levels in naive individuals and individuals who have recovered from COVID-19. Evaluation of anti-spike IgM (**A**), anti-S1 IgA (**B**), anti-spike (anti-S) IgG (**C**), anti-RBD IgG (**D**), spike-neutralizing Ig (**E**), and anti-nucleoprotein (anti-N) IgG (**F**) in naive individuals and individuals who have recovered from COVID-19 before the first injection, before the second injection, and at 1, 6, and 8 months after the complete vaccination cycle. Individual data from 15 naive individuals are shown on the left (red lines); individual data from 15 individuals who have recovered from COVID-19 are shown in the middle (blue lines); mean values ± SEM are shown on the right. Dashed lines represent cutoff values, dotted lines denote upper detection limits. Red and blue asterisks (left and middle) refer to paired statistics within each study group compared with the previous time point in the kinetics calculated with Wilcoxon’s signed-rank test. Red and blue pound signs (left and middle) denote paired statistics within each study group between baseline and 8 months, calculated with Wilcoxon’s signed-rank test. Black asterisks (right) denote unpaired statistics between naive individuals and individuals who have recovered from COVID-19 at each time point calculated with Mann-Whitney test. *P < 0.05; **P < 0.01; ***P < 0.001, ^###^*P <* 0.001.

As shown in Figure 1A, serum titers of anti-spike IgM increased in naive individuals (left panel) following the first vaccine administration, while they exhibited significant interindividual differences among individuals who have recovered from COVID-19. In both groups, anti-spike IgM declined from month 1, reaching values below the cutoff limit at months 6 and 8 (Figure 1A). Anti-spike IgA and anti-spike IgG peaked in naive individuals at month 1 after second dose administration, whereas in individuals who have recovered from COVID-19, they reached the highest levels after the first injection and remained stable up to month 1 (Figure 1, B and C, left and middle). Of note, these antibodies showed a mild decrease in SARS-CoV-2–experienced individuals at months 6 and 8 and a significant drop at month 6 in naive individuals. However, at month 8 anti-spike IgA and IgG displayed significant higher levels than those before vaccination in both groups. Anti–receptor binding domain (anti-RBD) IgG increased up to month 1, dropped significantly at month 6, and then remained at higher levels than baseline at month 8 in both groups (Figure 1D, left and middle). It should be noted that, at all time points of analysis, individuals who have recovered from COVID-19 showed significantly higher levels of anti-spike IgA and IgG and anti-RBD IgG than naive individuals (Figure 1, B–D, right). Neutralizing antibodies increased in naive individuals up to month 1 after vaccination, whereas they reached the highest level in individuals who have recovered from COVID-19 after the first injection, remaining stable up to month 1 (Figure 1E, left and middle). Despite a significant decrease at month 6, neutralizing antibodies declined less sharply than anti-RBD IgG in both groups (Figure 1E, right). Interestingly, neutralizing antibodies at month 8 were significantly higher than they were before vaccination in naive individuals, while we observed no significant difference in the recovered COVID-19 group. In addition, in this case, individuals who have recovered from COVID-19 showed higher antibody levels than naive individuals at all the analyzed time points.

Finally, individuals who have recovered from COVID-19 before vaccination displayed significantly higher levels of anti-nucleoprotein IgG than naive individuals, confirming their previous exposure to SARS-CoV-2 (Figure 1F). These antibodies decreased over time, reaching values below the cutoff limit at months 6 and 8. This finding confirms that also natural immunization to SARS-CoV-2 is subjected to decline over time. Naive individuals instead exhibited anti-nucleoprotein IgG below cutoff value at all time points (Figure 1F).

**Figure 2:**
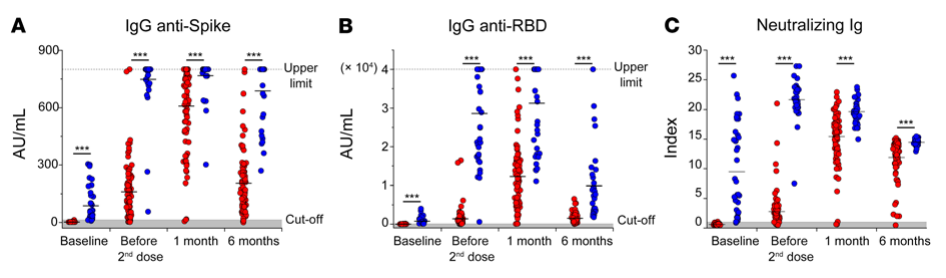
Longitudinal evaluation of anti-spike and neutralizing antibodies in a large cohort of naive individuals and individuals who have recovered from COVID-19. Evaluation of anti-spike IgG (**A**), anti-RBD IgG (**B**), and neutralizing Ig (**C**) before vaccination, before the second dose administration, and 1 and 6 months following the complete vaccination cycle in 86 naive individuals (red dots) and 39 individuals who have recovered from COVID-19 (blue dots). Black asterisks represent unpaired statistics between naive individuals and individuals who have recovered from COVID-19 at each time point calculated with Mann-Whitney test. ***P < 0.001.

Data obtained on an extended cohort of 86 naive individuals and 39 individuals who have recovered from COVID-19 confirmed that anti-spike IgG, anti-RBD IgG, and neutralizing Ig increase rapidly after the first vaccine injection in SARS-CoV-2–experienced individuals and then declined slowly up to month 6 after vaccination (Figure 2). On the contrary, in naive individuals, these antibodies peaked after the second dose administration, at month 1 after vaccination, but then sharply declined at month 6 (Figure 2). Notably, antibody levels in naive individuals did not reach the levels observed in recovered COVID-19 at all the analyzed time points (Figure 2).

### Naive individuals and individuals who have recovered from COVID-19 display comparable frequencies of spike-specific B cells at 8 months after vaccination.

**Figure 3:**
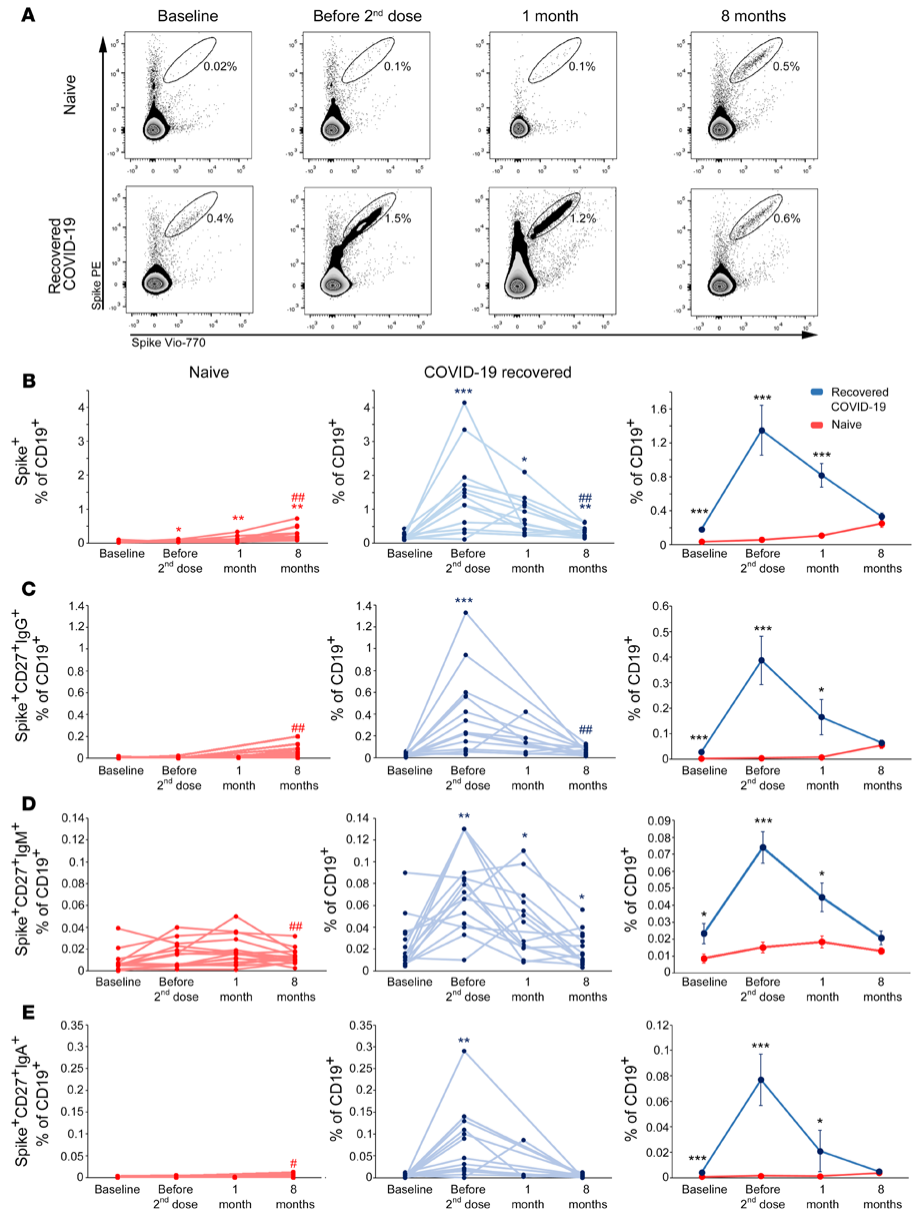
Longitudinal evaluation of spike-specific circulating B cells in naive individuals and individuals who have recovered from COVID-19. (**A**) Representative flow cytometry plots of spike-specific B cells in 1 naive individual (top) and 1 individual who has recovered from COVID-19 (bottom) before vaccination, before second injection, and after 1 and 8 months following the complete vaccination cycle. Longitudinal evaluation of frequencies of total (**B**), CD27^+^IgG^+^ (**C**), CD27^+^IgM^+^ (**D**) and CD27^+^IgA^+^ (**E**) spike-specific B cells in naive individuals (red lines, left) and individuals who have recovered from COVID-19 (blue lines, middle). Cumulative data are represented (right) as mean ± SEM from 15 naive individuals and 15 individuals who have recovered from COVID-19. Red and blue asterisks (left and middle) denote paired statistics within each study group compared with the previous time point in the kinetics calculated with Wilcoxon’s signed-rank test. Red and blue pound signs (left and middle) denote paired statistics within each study group between baseline and 8 months calculated with Wilcoxon’s signed-rank test. Black asterisks (right) represent unpaired statistics between naive individuals and individuals who have recovered from COVID-19 at each time point calculated with Mann-Whitney test. *P < 0.05; ^#^*P <* 0.05; **P < 0.01; ^##^*P <* 0.01; ***P < 0.001.

To further support the data obtained from the analysis of serum levels of SARS-CoV-2 spike-specific antibodies, we evaluated by flow cytometry the frequency of B cells expressing a B cell receptor (BCR) capable of recognizing SARS-CoV-2 spike protein (Figure 3A). As shown in Figure 3B, in naive individuals there was a gradual and significant increase in the frequency of B cells (CD19^+^) expressing a spike-specific BCR (spike^+^) up to month 8 following the administration of the second dose of vaccine. On the contrary, in individuals recovered from COVID-19, the significant increase in the frequency of spike-specific B cells observed following the first vaccine injection was followed by a progressive significant decline. Of note, at month 8 both naive individuals and individuals who have recovered from COVID-19 showed significantly higher levels of circulating spike-specific B cells than before vaccination. When comparing the frequency of these cells between the 2 study groups, we found that individuals who have recovered from COVID-19 exhibited significantly higher frequencies of circulating spike-specific B cells from baseline to month 1, while at month 8 we observed comparable percentages (Figure 3B, right). Circulating spike-specific B cells with a memory phenotype (CD27^+^) expressing on their surface a BCR of IgG, IgM, or IgA isotype exhibited a similar kinetics to the one shown by total spike-specific B cells in both groups (Figure 3, C–E, left and middle). In addition, these cells were significantly more represented in recovered COVID-19 than in naive individuals up to month 1 after vaccination, while they exhibited similar frequencies at month 8 (Figure 3, C–E, right).

### Frequency of spike-specific CD4^+^ T cells is comparable in naive individuals and individuals who have recovered from COVID-19 at month 1 after vaccination and it is stable up to month 8.

**Figure 4:**
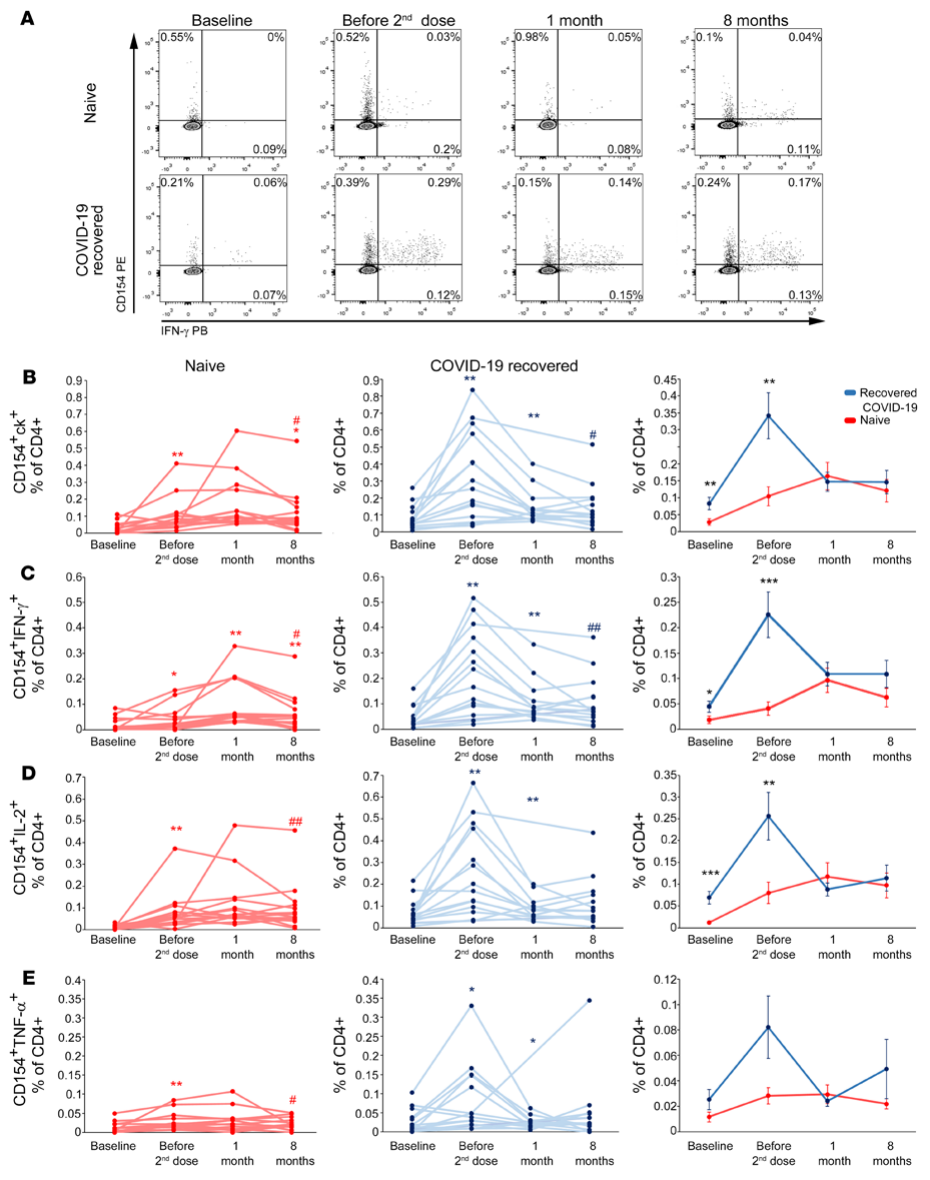
Longitudinal evaluation of spike-specific circulating T cells in naive individuals and individuals who have recovered from COVID-19. (**A**) Representative flow cytometric plots of spike-specific CD4^+^CD154^+^IFN-γ^+^ T cells in 1 naive individual (top) and 1 individual who has recovered from COVID-19 (bottom) before vaccination, before second injection, and after 1 and 8 months following the complete vaccination cycle. Longitudinal evaluation of frequencies of spike-specific T cells expressing CD154 and producing at least 1 cytokine (ck) among IL-2, IFN-γ, and TNF-α (**B**), CD154^+^IFN-γ^+^ (**C**), CD154^+^IL-2^+^ (**D**), and CD154^+^TNF-α^+^ (**E**) in naive individuals (red lines, left) and individuals who have recovered from COVID-19 (blue lines, middle). Cumulative data are represented (right) as mean ± SEM from 15 naive individuals and 15 individuals who have recovered from COVID-19. In **B**–**E**, data are shown after subtraction of the background, unstimulated condition. Red and blue asterisks (left and middle) refer to paired statistics within each study group compared with the previous time point in the kinetics calculated with Wilcoxon’s signed-rank test. Red and blue pound signs (left and middle) refer to paired statistics within each study group between baseline and 8 months, calculated with Wilcoxon’s signed-rank test. Black asterisks (right) represent unpaired statistics between naive individuals and individuals who have recovered from COVID-19 at each time point calculated with Mann-Whitney test. *P < 0.05; ^#^*P <* 0.05; **P < 0.01; ^##^*P <* 0.01; ***P < 0.001.

To obtain a complete picture of the adaptive immune response to anti–COVID-19 vaccination, we evaluated the antigen-specific CD4^+^ T cell response to SARS-CoV-2 spike protein peptides, monitoring CD154 expression and the production of IL-2, IFN-γ, and TNF-α upon in vitro stimulation (Figure 4A and Supplemental Figure 1). Total spike-specific CD4^+^ T cells, defined by CD154 expression and the production of at least 1 of the 3 analyzed cytokines (CD154^+^ck^+^), showed a gradual increase in naive individuals until month 1 after the second vaccine dose, followed by a slight decrease at month 8 (Figure 4B, left). On the contrary, in recovered individuals who had COVID-19, after an initial significant increase following the first vaccine dose, the frequency of these cells declined significantly at month 1 and then stabilized up to month 8. As observed for B cells, spike-reactive CD4^+^ T cells in both groups showed significantly higher frequencies at month 8 than before vaccination. In addition, total spike-specific CD4^+^ T cells were significantly higher in recovered individuals who had COVID-19 than in naive individuals until the administration of the second vaccine dose, while they became comparable at month 1 and remained similar also at month 8. These observations were confirmed also when focusing on CD4^+^ T cells specifically secreting IFN-γ (Figure 4C), IL-2 (Figure 4D), or TNF-α (Figure 4E).

**Figure 5:**
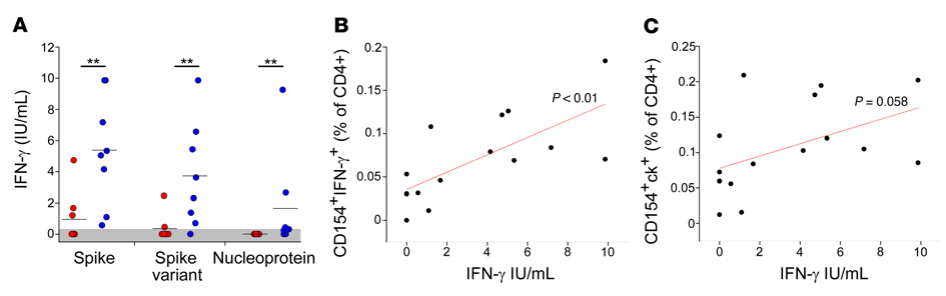
Quantification of soluble IFN-γ levels following stimulation with SARS-CoV-2 antigens by naive individuals and individuals who have recovered from COVID-19. (**A**) Levels of IFN-γ in sera following whole-blood stimulation with spike (from ancestral strain and Alpha variant), spike variant (from Beta and Gamma variants), or nucleoprotein performed on 8 naive individuals (red dots) and 8 individuals who have recovered from COVID-19 (blue dots) at month 8 after vaccination. Represented data are subtracted of background, unstimulated condition. (**B**) Correlation between the frequency of circulating CD4^+^CD154^+^IFN-γ^+^ cells and IFN-γ serum levels following whole stimulation with spike. (**C**) Correlation between the frequency of circulating CD4^+^CD154^+^ cells producing at least 1 cytokine (among IFN-γ, IL-2, and TNF-α) and IFN-γ serum levels following whole-blood stimulation with spike. Pearson’s *r* was used for correlations. **P < 0.01.

To characterize more deeply the effector capacity of vaccine-induced CD4^+^ T cells, we also monitored the amount of IFN-γ secreted by cells collected at month 8 after vaccination and stimulated overnight with SARS-CoV-2 antigens. T cells from individuals who have recovered from COVID-19 produced significantly higher levels of IFN-γ following stimulation with spike, if compared with those of naive individuals (Figure 5A). Of note, the same difference was observed even when stimulating with spike variants (Beta and Gamma) (Figure 5A). As expected, IFN-γ was detected only in individuals who have recovered from COVID-19 following stimulation with nucleoprotein, although in only 5 of 8 tested individuals (Figure 5A). Interestingly, IFN-γ levels detected in the serum following spike peptide pool stimulation directly correlated with the frequency of CD154^+^IFN-γ^+^ spike-specific CD4+ T cells (Figure 5B). While still showing this trend, the strength of this correlation was reduced below the significance cutoff when correlating IFN-γ production with the frequency of total spike-specific CD4^+^ T cells, suggesting that IFN-γ secretion is not a characteristic feature of all spike-specific CD4^+^ T cells. (Figure 5C).

### Booster vaccination reactivates humoral and cellular immunity in naive individuals more efficiently than in individuals who have recovered from COVID-19.

**Figure 6:**
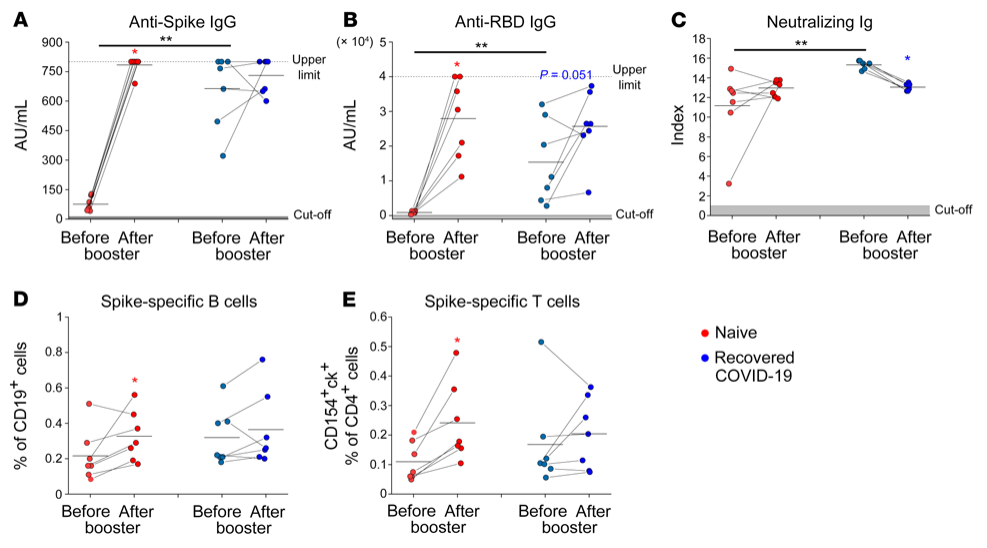
Reactivation of humoral and cellular immunity following vaccine booster administration in naive individuals and individuals who have recovered from COVID-19. Levels of anti-spike IgG (**A**), anti-RBD IgG (**B**), neutralizing Ig (**C**) in 7 naive individuals (red) and 7 individuals who have recovered from COVID-19 (blue) before (pre) and 1 week after (post) vaccine booster injection. Levels of spike-specific B (**D**) and CD4^+^ T (**E**) (defined as CD154^+^ and producing at least 1 cytokine among IFN-γ^+^, IL-2, and TNF-α cells) in 7 naive individuals (red) and 7 individuals who have recovered from COVID-19 (blue) before and 1 week after vaccine booster injection. Red and blue asterisks denote paired statistics within each study calculated with Wilcoxon’s signed-rank test. Black asterisks (right) represent unpaired statistics between naive individuals and individuals who have recovered from COVID-19 calculated with Mann-Whitney test. *P < 0.05; **P < 0.01

The progressive decline in vaccine efficacy observed 6 months after the second dose administration led many countries to the decision to proceed with the administration of a third dose of vaccine (booster dose). In this regard, we had the opportunity to evaluate humoral, B and CD4^+^ T cell–mediated spike-specific immune response in 14 (7 naive and 7 recovered from COVID-19) individuals before and 1 week after the administration of the booster dose. We evaluated the immune response 1 week after booster administration, because we have previously demonstrated that 1 week after first vaccine administration there is the maximal reactivation of anti-spike immunity in individuals who have recovered from COVID-19 (3). As shown in Figure 6, A and B, serum titers of anti-spike IgG and anti-RBD IgG significantly increased in naive individuals but not in individuals who have recovered from COVID-19 after the booster dose. Interestingly, while serum titers of spike neutralizing Ig were not affected by the booster dose in naive individuals, a significant reduction was induced 1 week after the administration in individuals who have recovered from COVID-19 (Figure 6C). Despite a significantly different amount of anti-spike IgG, anti-RBD IgG, and neutralizing Ig before the booster, the 2 study groups showed comparable amounts of these antibodies 1 week after the injection. In agreement with antibody data, the frequencies of spike-specific B cells and CD4^+^ T cells significantly increased in naive individuals but not in individuals who have recovered from COVID-19 after the booster administration (Figure 6, D and E). In this case as well, spike-specific B and CD4^+^ T cells exhibited comparable frequencies between the 2 groups after booster.

### Antibodies, but not B and CD4^+^ T cells, decline up to 1 year following natural infection.

**Figure 7:**
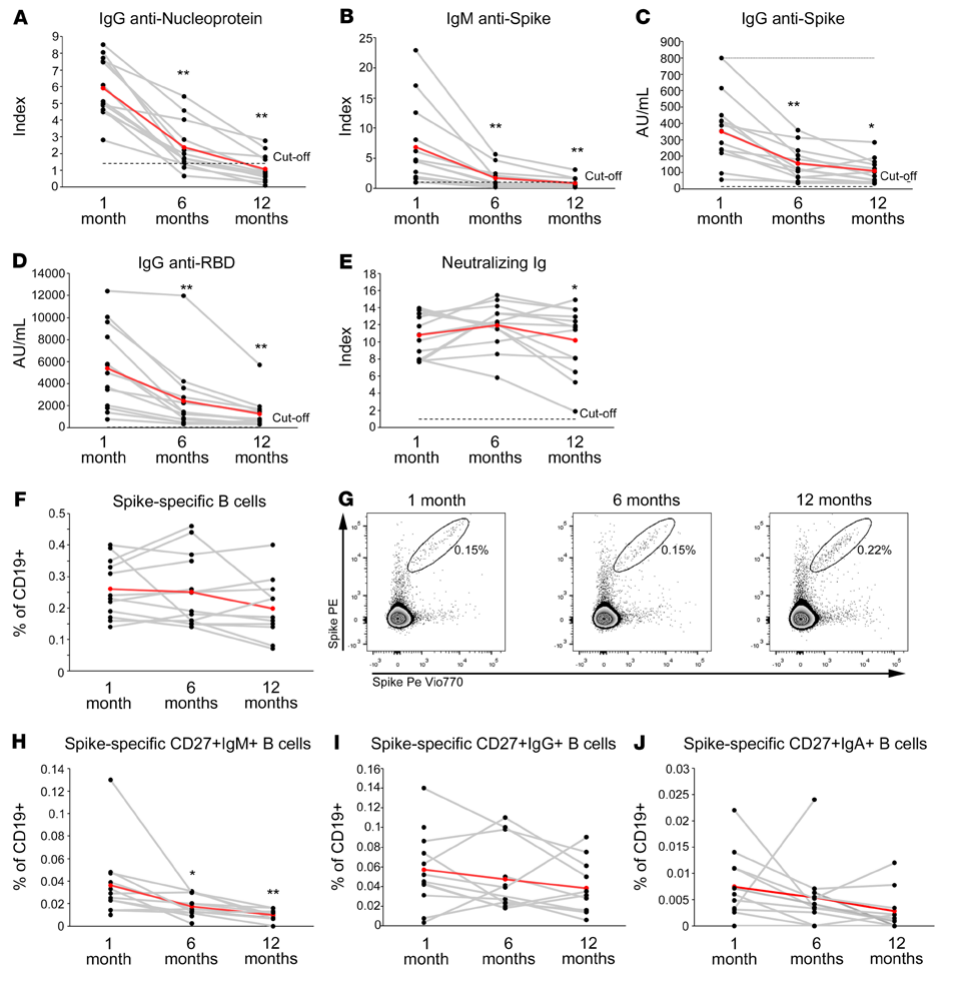
Longitudinal evaluation of SARS-CoV-2–specific antibody levels up to 1 year following infection. Levels of anti-nucleoprotein IgG (**A**), anti-spike IgM (**B**), anti-spike IgG (**C**), anti-RBD IgG (**D**), and neutralizing Ig (**E**) in 13 individuals who have recovered from COVID-19 evaluated 1, 6, and 12 months following hospital discharge. Frequencies of total (**F**), CD27^+^IgM^+^ (**H**), CD27^+^IgG^+^ (**I**), CD27^+^IgA^+^ (**J**) spike-specific B cells evaluated at 1, 6, and 12 months following hospital discharge in 14 individuals who have recovered from COVID-19. Representative FACS plots of total spike-specific B cells at each time point are shown in **G**. Red lines represent mean values. Asterisks refer to paired statistics at each time point compared with the previous, calculated with Wilcoxon’s signed-rank test. *P < 0.05; **P < 0.01.

In order to compare the longevity of immunological memory induced by vaccination or SARS-CoV-2 natural infection, we enrolled 14 unvaccinated individuals who have recovered from COVID-19 who were longitudinally sampled at 1, 6, and 12 months following hospital discharge. Because vaccine administration is recommended in individuals who have recovered from COVID-19, this is a rather unique cohort. We evaluated serum levels of anti-nucleoprotein IgG, anti-spike IgM and IgG, anti-RBD IgG, and spike neutralizing Ig as well as the frequencies of spike-specific B and CD4^+^ T cells. As shown in Figure 7, anti-nucleoprotein IgG, anti-spike IgM and IgG, and anti-RBD IgG significantly declined at month 6 and even more so at month 12 after hospital discharge (Figure 7, A–D), whereas spike-neutralizing Ig levels remained stable over time (Figure 7E).

**Figure 8:**
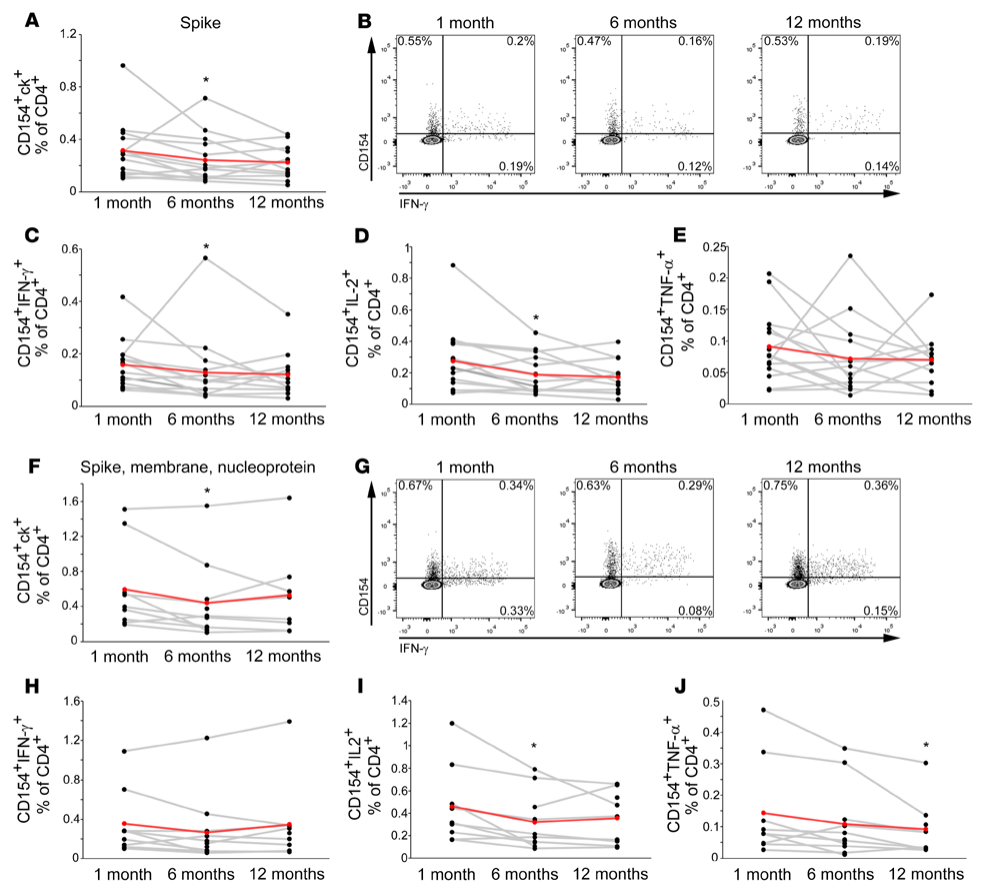
Longitudinal evaluation of SARS-CoV-2–specific B and T cells up to 1 year following infection. Frequencies of spike-specific CD4^+^ T cells expressing CD154 and producing at least 1 cytokine among IL-2, IFN-γ, and TNF-α (**A**), CD154^+^IFN-γ^+^ (**C**), CD154^+^IL-2^+^ (**D**), and CD154^+^TNF-α^+^ (**E**) evaluated at 1, 6, and 12 months following hospital discharge in 14 individuals who have recovered from COVID-19. Representative FACS plots of CD154^+^IFN-γ^+^ spike-specific CD4+ T cells a at each time point are shown in **B**. Frequencies of CD4^+^ T cells reactive to spike, membrane, and nucleoprotein expressing CD154 and producing at least 1 cytokine among IL-2, IFN-γ and TNF-α (**F**), CD154^+^IFN-γ^+^ (**H**), CD154^+^IL-2^+^ (**I**), and CD154^+^TNF-α^+^ (**J**) evaluated at 1, 6, and 12 months following hospital discharge in 14 individuals who have recovered from COVID-19. Representative FACS plots of CD154^+^IFN-γ^+^ CD4+ T cells reactive to spike, membrane, and nucleoprotein at each time point are shown in **G**. Red lines represent mean values. Asterisks denote paired statistics at each time point compared with the previous, calculated with Wilcoxon’s signed-rank test. *P < 0.05.

Prominently, the frequency of circulating B cells expressing a BCR capable of recognizing SARS-CoV-2 spike protein was substantially comparable over time (Figure 7, F and G). Moreover, with the exception of spike-specific CD27^+^ IgM B cells, the frequencies of CD27^+^IgG^+^ and CD27^+^IgA^+^ spike-specific B cells did not decline (Figure 7, H–J). Similarly, the frequency of SARS-CoV-2–specific CD4^+^ T cells, as assessed by the expression of CD154 and the ability to produce at least 1 of the cytokines IL-2, IFN-γ, or TNF-α upon in vitro stimulation with spike or combination of spike, nucleoprotein, and membrane peptides pools, was characterized by stability over time (Figure 8, A, B, F, and G). The same stability was observed even when evaluating the production of specific cytokines (IFN-γ, IL-2, or TNF-α), in response to spike or to the combination of spike, membrane, and nucleoprotein (Figure 8, C–E, and H–J).

## Discussion

Anti–COVID-19 vaccines have shown remarkable efficacy, both in phase III clinical trials and in real-world studies, demonstrating protection against severe disease and infection (12–14). Vaccination leads to the development of humoral and cellular immunity against spike (3, 10). However, it is currently unknown how long immunological memory induced by vaccination will last, with fundamental implications for vaccination strategies and the future course of the current pandemic. Epidemiological data have shown that 6 months following the complete 2-doses mRNA vaccination cycle, vaccine effectiveness against infection drops down from 88% to 47% (7). Nonetheless, protection against severe disease remains high when compared with that in unvaccinated people (8). The reduced efficacy over time may be the result of the progressive waning of immunological memory, the occurrence of new SARS-CoV-2 variants with increased transmissibility and immune evasion potential, or a combination of both. In this study, we investigated the longevity of vaccine-induced humoral and cellular immunity to spike up to 8 months following vaccination in naive individuals and individuals who have recovered from COVID-19. We and others have previously shown that, in individuals who have recovered from COVID-19, 1 dose of mRNA vaccine is sufficient to achieve high levels of anti-spike immunity, higher than those observed in naive individuals after 2 vaccine injections (3, 8). In line with this observation, we found here that anti-spike antibodies are maintained at higher levels in individuals who have recovered from COVID-19 at months 6 and 8 after vaccination. Indeed, anti-spike IgG and anti-RBD IgG showed a significant drop from month 1 to month 6 in naive individuals. However, the observed reduction in total anti-spike antibodies does not necessarily reflect a loss in the quality of humoral response. Indeed, affinity maturation may select B cells producing antibodies with improved antigen-binding capability, thus maintaining the overall functionality despite a reduction in total antibody levels. In agreement with this possibility, neutralizing antibodies declined less rapidly in both groups. This peculiar kinetics suggests that affinity maturation occurs over time. These findings are in agreement with those of a recent publication, showing that neutralizing antibodies decline slower than total anti-spike Ig in both naive individuals and individuals who have recovered from COVID-19 (15). In contrast to antibody levels, we observed that anti-spike memory cells are comparable at month 8 between naive individuals and individuals who have recovered from COVID-19. Regarding spike-specific B cells, we found that the initial massive expansion observed early after the first vaccination in individuals who have recovered from COVID-19 was followed by a significant contraction phase. On the contrary, in naive individuals spike-specific B cells were very rare at initial time points but then increased over time from month 1 to month 8. Spike-specific CD4^+^ T cells instead showed comparable frequency in the 2 groups at month 1 and then levels slowly declined in parallel up to month 8. Regarding the effector capacity, we observed that despite similar frequencies of vaccine-induced CD4^+^ T cells at month 8, individuals who have recovered from COVID-19 produced significantly more IFN-γ following stimulation with spike than naive individuals. Of note, this observation was confirmed when stimulating with SARS-CoV-2 spike from Beta and Gamma variants, confirming previous data showing that the vaccine-induced T cell response is largely conserved against viral variants (16–18). It should be noted that the frequencies of spike-specific B and CD4^+^ T cells at month 8 are at least comparable to those observed in individuals who have recovered from COVID-19 before vaccination, confirming that they are still detectable at this time point. These kinetics are similar to those observed by Goel et al., who monitored the persistence of spike-specific memory cells immunity up to 6 months following vaccination (15).

The picture that comes from these observations shows that antibody levels decline rapidly a few months after vaccination, while memory B and CD4^+^ T cells display features of long-term persistence. This scenario is compatible with the loss of vaccine efficacy observed in real-world studies 6 months after vaccination (7). Indeed, the progressive waning of antibody levels, coupled with the reduced neutralization efficacy of vaccinees’ sera against Delta and Omicron variants, may expose individuals to an increased risk of infection (19–22). Indeed, many breakthrough infections have been reported worldwide, even after the booster injection (23), in individuals with likely high antibody titers. This finding means that the escape capability of the new viral variant is predominant over the antibody decline in favoring the infection. However, the presence of memory B and CD4^+^ T cells, which can rapidly reactivate following the encounter with SARS-CoV-2, can be a reason for the retained efficacy against severe disease.

The administration of a third (booster) vaccine injection has shown to restore protection against symptomatic disease (24, 25). For this reason, given the increased numbers of breakthrough infections, several countries have initiated booster campaigns. We monitored the effect of a third-dose injection in a cohort of naive and SARS-CoV-2–experienced individuals. Our results showed that the booster dose significantly increased the levels of spike-specific antibodies and B and CD4^+^ T cells in naive individuals. This observation provides the immunological reason for the restored efficacy against symptomatic COVID-19. Longitudinal studies are needed to understand if a booster injection will lead to sustained maintenance of anti-spike humoral immunity, or whether a similar decline will occur in the coming months. In this case, it is plausible that regular boosters may be needed to keep vaccine efficacy high. The observation that SARS-CoV-2 infection followed by 2 vaccine injections results in longer maintenance of anti-spike antibodies suggests that the third vaccine dose may result in longer antibody persistence in those unexposed to SARS-CoV-2. In contrast to that observed in naive individuals, the third vaccine dose has only minor effects on individuals who have recovered from COVID-19, in line with what was observed for the second vaccine dose (3, 10). Given that at month 8 these individuals displayed high anti-spike antibody levels, as well as presence of memory B and CD4^+^ T cells, regulatory mechanisms may be involved in preventing an overactivation of anti-spike immunity. On the other hand, it may be possible that spike-specific B and CD4^+^ T cells display an exhausted phenotype, which prevents optimal reactivation upon antigen restimulation. Additional studies are needed to fully understand this phenomenon. However, this observation raises the question about the need for boosters in individuals who have recovered from COVID-19.

In this manuscript, we also monitored the long-term persistence of immunity to SARS-CoV-2 up to 1 year following infection in a group of individuals recovered from moderate-to-severe COVID-19. This is a unique cohort, as in the majority of countries vaccination has been recommended to individuals who have a history of SARS-CoV-2 infection. The data obtained are largely comparable to those observed following vaccination, with a progressive decline in antibody levels. Anti-nucleoprotein IgG displayed the shortest persistence, as its levels dropped below the cutoff value at month 12. Notably, as in the case of natural infection, neutralizing antibodies decayed slower than total anti-spike IgG and anti-RBD IgG. The progressive reduction of antibody levels may be the reason for the observed reinfections (26, 27). On the contrary, the frequencies of spike-specific B and CD4^+^ T cells were largely stable up to 1 year from hospital discharge. The same stability was observed when expanding our analysis to other SARS-CoV-2 antigens, as the frequency of CD4^+^ T cells reactive to spike, membrane, and nucleoprotein was basically stable from month 1 to month 12. Collectively, these findings suggest that people recovering from moderate-to-severe COVID-19 may develop long-term persistent memory cells to SARS-CoV-2, in agreement with previous observations obtained on the original SARS-CoV showing that memory cells can be detected up to 17 years after infection (28). This conclusion may not be extended to all SARS-CoV-2–infected individuals, as previous data have shown that asymptomatic infection leads to the development of reduced levels of immunological memory to the virus (5, 29).

In conclusion, our data show that vaccine- and infection-induced immunological memory display a similar kinetics of decay. Short-term persistence of humoral immunity may be responsible of reinfections, although long-lived memory B and CD4^+^ T cells may protect from severe disease development. A booster dose is useful in naive individuals, as it increases anti-spike immunity, reaching the levels observed in the recovered COVID-19 cohort. In this latter group, on the contrary, an additional injection is ineffective and, thus, unnecessary.

## Methods

### Individuals.

30 healthcare workers who received the BNT162b2 mRNA COVID-19 vaccine were recruited at the Careggi University Hospital by the Infectious and Tropical Diseases Unit. Among them, 15 individuals had a previous history of symptomatic SARS-CoV-2 infection (recovered COVID-19). Confirmation of SARS-CoV-2 infection was obtained by PCR analysis of nasopharyngeal swab. Negative history for infection in naive individuals was based on absence of symptoms, absence of anti-nucleoprotein and anti-spike IgG before receiving vaccination, and routine monitoring by nasopharyngeal swab PCR testing. Among individuals who have recovered from COVID-19, disease severity was defined according to WHO guidelines (30). Basic demographic and clinical characteristics are summarized in Supplemental Table 1. Following immunization schedule originally approved by the European Medicines Agency, each individual received 2 vaccine injections, 21 days apart. Blood samples were collected before the first dose (basal time), before the second dose, and then 1 month, 6 months, and 8 months later. 95 additional individuals (71 naive and 24 recovered COVID-19) were also recruited in addition to the 30 aforementioned individuals to expand serological data on a larger cohort. These individuals (125 in total, 86 naive and 39 recovered COVID-19) were serologically tested before the first injection, before the second injection, and then 1 month and 6 months later. Recruited individuals were not affected by chronic medical conditions that may affect vaccine response, with the exception of only 1 individual in the recovered COVID-19 group, who was under immunosuppressive treatment following solid organ transplantation. Basic demographic and clinical characteristics of the expanded cohort are summarized in Supplemental Table 2. 14 individuals, 7 of whom had previous SARS-CoV-2 infection, were sampled before booster BNT162b2 injection and 1 week after. All individuals received the booster dose 9 months after the second vaccine dose. Basic demographic and clinical characteristics are summarized in Supplemental Table 3.

Peripheral blood samples from 14 individuals hospitalized for COVID-19 between March and April 2020 were obtained 1 month, 6 months, and 12 months following discharge. These individuals were not vaccinated at the last follow-up date. Basic demographic and clinical characteristics are summarized in Supplemental Table 4.

PBMCs were obtained following density gradient centrifugation of blood samples using Lymphoprep (Axis Shield Poc As) and were frozen in FCS plus 10% DMSO for storage in liquid nitrogen. For each individual, longitudinal samples were defrosted and analyzed together for B cell and T cell evaluation. Serum was frozen and stored for Ig levels evaluation.

### Evaluation of SARS-CoV-2 spike-reactive T cells.

For T cell stimulation in vitro, 1.5 million PBMCs were cultured in complete RPMI plus 5% human AB serum in 96-well flat-bottom plates in presence of medium alone (background, negative control) or of a pool of spike SARS-CoV-2 peptide pools (Prot_S1, Prot_S+, and Prot_S to achieve a complete sequence coverage of the spike protein) at 0.6 μM/peptide, accordingly to manufacturer’s instructions (Miltenyi Biotech). PBMCs from individuals who have recovered from COVID-19 longitudinally assessed up to 1 year following hospital discharge were additionally stimulated with peptide pools of membrane protein and nucleoprotein from SARS-CoV-2 at 0.6 μM/peptide, accordingly to manufacturer’s instructions (Miltenyi Biotech). After 2 hours of incubation at 37°C, 5% CO_2_, brefeldin A (5 μg/mL) was added, followed by additional 4-hour incubation. Finally, cells were fixed and stained using the fluorochrome-conjugated antibodies listed in Supplemental Table 5. Samples were acquired on a BD LSR II flow cytometer (BD Biosciences) with FACSDiva Software and analyzed by FlowJo Software. The gating strategy used is shown in Supplemental Figure 2.

### Evaluation of SARS-CoV-2 spike-specific B cells.

For B cell evaluation, 2 million PBMCs were stained for 30 minutes at 4°C with fluorochrome-conjugated antibodies listed in Supplemental Table 6, then washed with PBS/EDTA buffer, and incubated for 5 minutes with 7-AAD for viability evaluation. Recombinant biotinylated SARS-Cov2 spike protein (Miltenyi Biotech) was conjugated separately with streptavidin PE and PE-Vio770 for 15 minutes at room temperature and pooled in 1:2 ratio, before being added to final staining mix. Samples were acquired on a BD LSR II flow cytometer (BD Biosciences) with FACSDiva Software and analyzed by FlowJo Software. The gating strategy used is shown in Supplemental Figure 3.

### Evaluation of SARS-CoV-2–specific IgA, IgM, and IgG.

Evaluation of SARS-CoV-2 spike protein antibodies, including anti-spike-specific (in trimeric form) IgG (Diasorin), anti-spike RBD-specific IgG (Abbott) and IgA (Euroimmun), anti-spike-specific IgM (Abbot), anti-nucleoprotein-specific IgG (Abbott), and neutralizing antibodies that block binding of spike protein with the ACE2 receptor (Dia.Pro Diagnostic Bioprobes), was performed following manufacturers’ instructions. The antibody reactivity of each specimen was expressed in AU/ml or by the ratio between optical density and cutoff value (index).

### Evaluation of IFN-γ in culture supernatants following blood stimulation with SARS-CoV-2 antigens.

Evaluation of IFN-γ in culture supernatants following blood stimulation with SARS-CoV-2 antigens was performed with the STANDARD F Covi-FERON FIA (IFN-γ) SD Biosensor according to the manufacturer’s instructions. Viral antigens used for stimulation included SARS-CoV-2 spike protein antigens derived from the original SARS-CoV-2 strain and the 20I/501Y.V1 (Alpha), 20H/501 (Beta), and 20J/501Y.V3 (Gamma) viral variants as well as antigens derived from the nucleocapsid protein. The results were expressed as IU/ml of IFN-γ, and unstimulated background conditions were subtracted.

### Statistics.

Unpaired Mann-Whitney test was used to compare individuals who have recovered from COVID-19 and naive individuals; Wilcoxon’s signed-rank test was used to compare different time points in each study group. In all cases, *P* values of less than or equal to 0.05 were considered significant.

### Study approval.

The procedures followed in the study were approved by the Careggi University Hospital Ethical Committee. Written informed consent was obtained from recruited patients.

## Author contributions

GMR, AB, and FA, designed the study. NDL, EM, MS, LZ, and F Lagi collected peripheral blood samples and obtained informed consent. F Lagi and LC provided advice. AM, LM, LS, AV, MC, GL, STK, MGC, AR, AC, and PF, performed experiments. AM and LM, analyzed data. AM, LM, and FA wrote the manuscript. All authors revised the manuscript and gave final approval.

## Supplementary Material

Supplemental data

ICMJE disclosure forms

## Version 1 02/09/2022

In-Press Preview

## Version 2 03/15/2022

Electronic publication
