# Supplementary material for: SARS-CoV-2 infection and vaccination trigger long-lived B and CD4+ T lymphocytes with implications for booster strategies
Source: J Clin Invest. 2022 Mar 15;132(6):e157990. doi: 10.1172/JCI157990 (PMC8920339; doi:10.1172/JCI157990)
Supplement: Supplemental data [file jci-132-157990-s081.pdf]

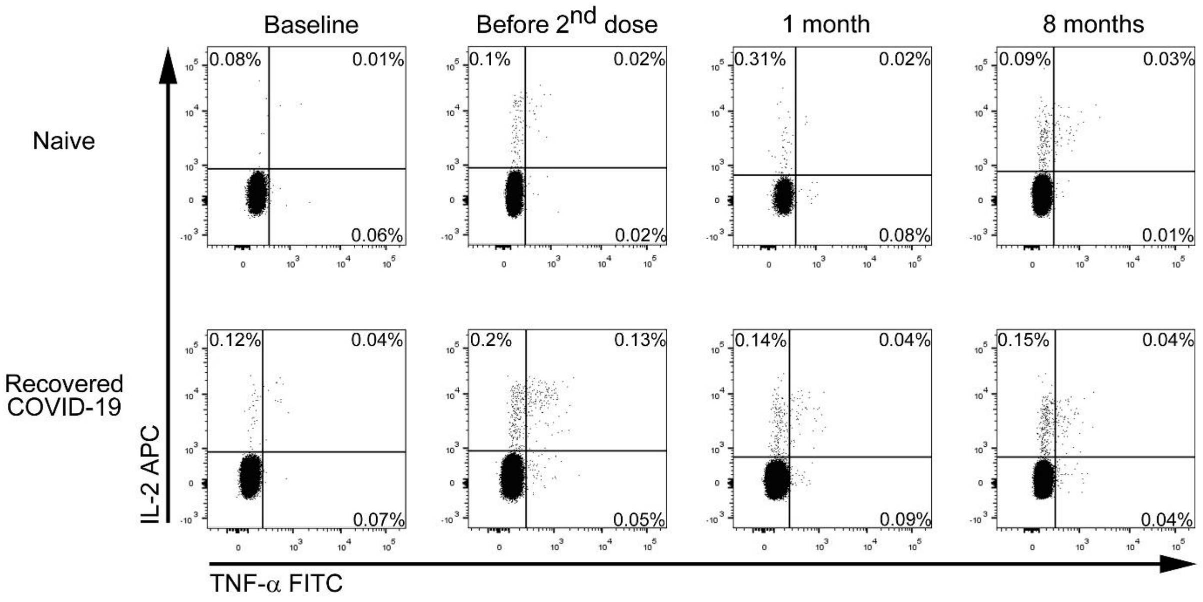

Supplemental Figure 1

Representative flow cytometric plots of Spike-specific CD4+ IL-2+ TNF-α+ T cells in one naïve (upper row) and one COVID-19 recovered (lower row) subject before vaccination, before second injection and after 1 and 8 months following the complete vaccination cycle.

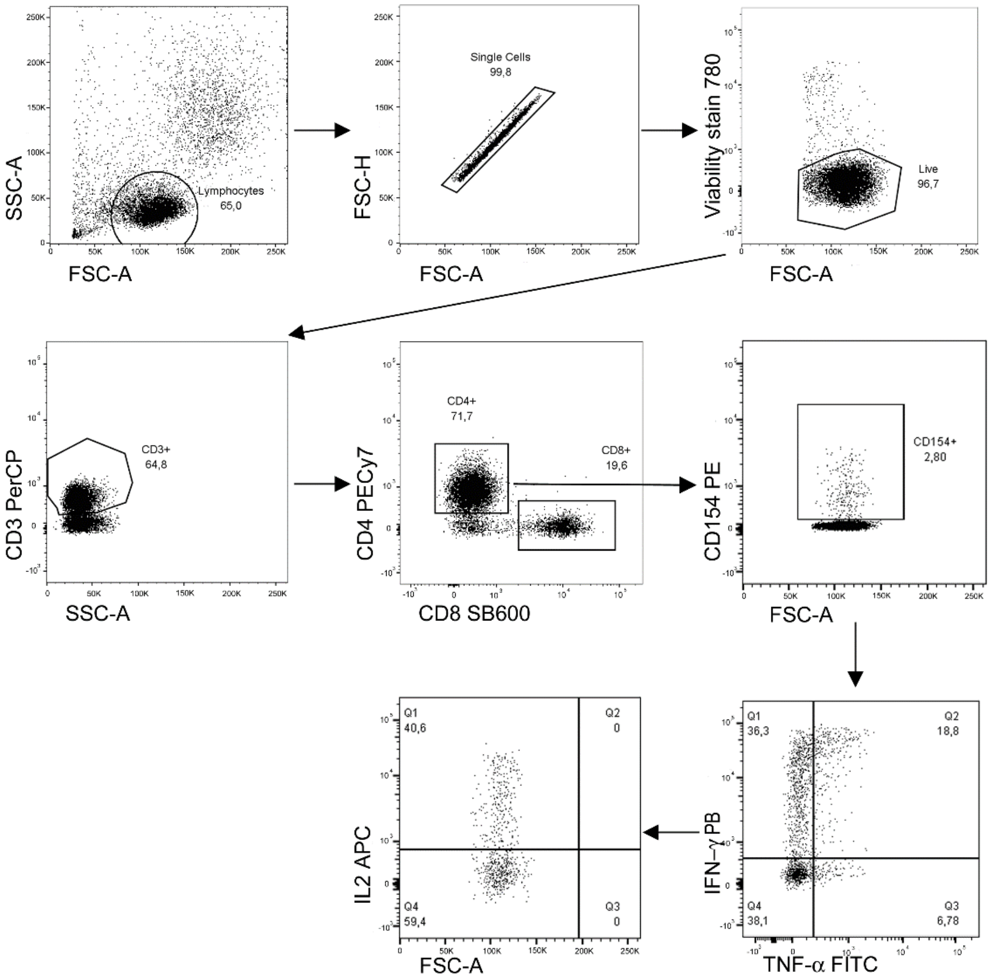

Supplemental Figure 2. Gating strategy for the identification of spike-specific CD4+ T cells

Lymphocytes were gated based on physical parameters (FSC-SSC), then doublets were removed using FSC-A and FSC-A parameters. Dead cells were excluded using viability stain 780. T cells were identified as CD3+. We then identified CD4+ T cells. Among these, we identified CD154 expressing cells. CD4+CD154+ T cells were then evaluated for IFN-γ, TNF-α and IL-2 expression.

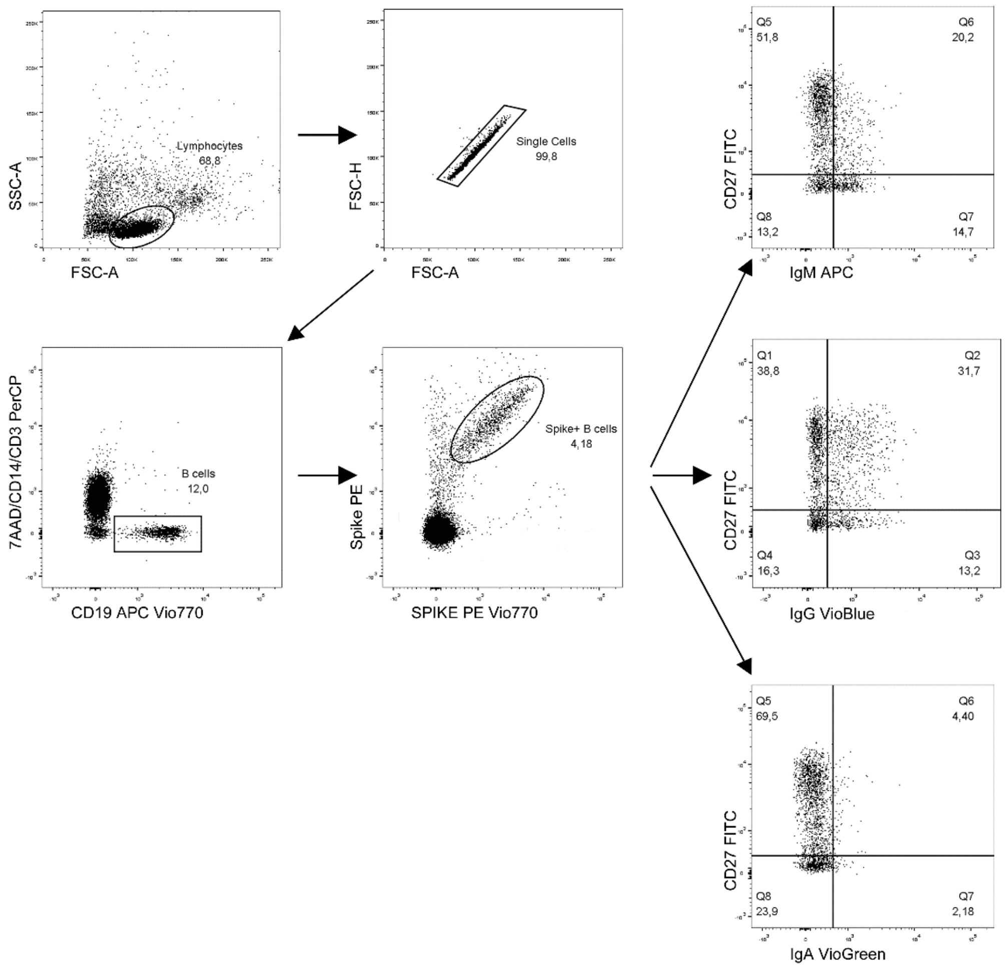

Supplemental Figure 3: Gating strategy for the identification of spike-specific B cells

Lymphocytes were gated based on physical parameters (FSC-SSC), then doublets were removed using FSC-A and FSC-A parameters. PerCP was used as dump channel for the exclusion of dead cells (7AAD), CD3+ T cells and CD14 monocytes. B cells were identified as CD19+. B cells binding PE- and PE Vio770-conjugated spike protein were then identified as spike-specific. Among spike-specific B cells we evaluated CD27 expression associated to IgG, IgM and IgA.

Supplemental Table S1. Basic demographic and clinical data of 30 healthcare workers who received BNT162b2 mRNA COVID-19 vaccine and were followed up to 8 months.

	Subject ID	Age (years)	Gender	COVID-19 severity	Time from COVID-19 diagnosis° to first vaccine dose (days)
COVID-19 recovered individuals	R1	46	male	mild	214
	R2	35	female	mild	58
	R3	51	female	severe	294
	R4	52	female	mild	286
	R5	60	male	moderate	302
	R6	65	male	mild	312
	R7	53	female	mild	289
	R8	37	male	moderate	297
	R9	54	female	mild	293
	R10	29	female	mild	289
	R11	39	male	mild	72
	R12	61	male	critical	288
	R13	64	male	critical	292
	R14	51	female	mild	222
	R15	44	female	mild	306
Mean±SD	-	49±11	-	-	254±82
Naïve individuals	N1	63	male	na	na
	N2	41	female	na	na
	N3	32	male	na	na
	N4	44	female	na	na
	N5	35	female	na	na
	N6	61	female	na	na
	N7	41	male	na	na
	N8	37	female	na	na
	N9	32	male	na	na
	N10	28	male	na	na
	N11	34	male	na	na
	N12	29	female	na	na
	N13	46	female	na	na
	N14	24	female	na	na
	N15	53	male	na	na
Mean±SD	-	40±12	-	-	-

na denotes *not applicable*
°first positive test on nasopharyngeal swab

Supplemental Table S2. Basic demographic and clinical data of 125 healthcare workers who received BNT162b2 mRNA COVID-19 vaccine and were followed up to 6 months.

	**COVID-19 recovered individuals**	**Naïve individuals**
**No. subjects**	39	86
**Age** (years)<br>Mean±SD	50.4±11.3	45.5±14.3
**Gender**<br>- Male, n (%)<br>- Female, n (%)	12 (31.6)<br>26 (68.4)	39 (45.3)<br>47 (54.6)
**COVID-19 severity**<br>- Asymptomatic/mild, n (%)<br>- Moderate, n (%)<br>- Severe, n (%)<br>- Critical, n (%)	31 (79.5)<br>2 (5.1)<br>2 (5.1)<br>4 (10.3)	na
**Time from COVID-19 diagnosis° to first vaccine dose (days)**<br>Mean±SD	230.4±93.6	na

na denotes *not applicable*
°first positive test on nasopharyngeal swab

Supplemental Table S3. Basic demographic and clinical data of 14 healthcare workers who received booster BNT162b2 mRNA COVID-19 vaccine injection and were followed up to one week.

	**Subject ID**	**Age (years)**	**Gender**	**COVID-19 severity**
**COVID-19 recovered individuals receiving booster dose**	RB1	52	female	severe
	RB2	53	female	mild
	RB3	65	male	critical
	RB4	37	male	moderate
	RB5	55	female	mild
	RB6	62	male	critical
	RB7	54	female	mild
Mean±SD	-	54±9	-	-
**Naïve individuals receiving booster dose**	NB1	45	female	na
	NB2	56	female	na
	NB3	36	male	na
	NB4	36	female	na
	NB5	29	male	na
	NB6	31	male	na
	NB7	42	male	na
Mean±SD	-	39±9	-	-

na denotes *not applicable*

Supplemental Table S4. Basic demographic and clinical data of 14 individuals who were hospitalized for COVID-19 between March and April 2020 and followed up to 12 months from discharge.

	Subject ID	Age (years)	Gender	COVID-19 severity
Unvaccinated COVID-19 recovered individuals	U1	56	male	moderate
	U2	61	male	moderate
	U3	58	male	severe
	U4	75	female	severe
	U5	48	male	moderate
	U6	62	male	severe
	U7	58	male	critical
	U8	73	male	critical
	U9	73	female	severe
	U10	56	female	critical
	U11	56	female	moderate
	U12	54	female	moderate
	U13	57	female	moderate
	U14	69	male	critical
Mean±SD	-	61±8	-	-

Supplemental Table S5. List of all fluorochrome mAbs used for flow cytometric analysis of antigen specific T cells.

**Antigen**	**Flurochrome**	**Clone**	**Company**
TNF-α	FITC	6401.1111	BDBioscience
CD154	PE	TRAP1	BDBioscience
CD3	PerCP	SK7	BDBioscience
CD4	PE-Cy7	SK3	Invitrogen
CD8	Super Bright 600	SK1	eBioscience™
IL-2	APC	MQ1-17H12	BDBioscience
IFN-γ	Pacific Blue	B27	BioLegend
L/D	Fixable Viability Stain 780		BDBioscience

Supplemental Table S6. List of all fluorochrome mAbs used for flow cytometric analysis of antigen specific B cells.

Antigen	Flurochrome	Clone	Company
CD19	APC-Vio770	LT19	Miltenyi Biotech
CD27	VioBright FITC	M-T271	Miltenyi Biotech
IgA	VioGreen	IS11-8E10	Miltenyi Biotech
IgG	VioBlue	IS11-3B2.2.3	Miltenyi Biotech
CD14	PerCP	TÜK4	Miltenyi Biotech
IgM	APC	PJ2-22H3	Miltenyi Biotech
CD3	PerCP	BW264/56	Miltenyi Biotech
7AAD			Miltenyi Biotech
Spike	PE		Miltenyi Biotech
Spike	PE-Vio770		Miltenyi Biotech
